# Primary Cilium-Dependent Signaling Mechanisms

**DOI:** 10.3390/ijms18112272

**Published:** 2017-10-28

**Authors:** Rajasekharreddy Pala, Nedaa Alomari, Surya M. Nauli

**Affiliations:** 1 Department of Biomedical & Pharmaceutical Sciences, Chapman University, Irvine, CA 92618, USA; rrpala@chapman.edu (R.P.); aloma112@mail.chapman.edu (N.A.); 2 Harry and Diane Rinker Health Science Campus, Chapman University, 9401 Jeronimo Road, Irvine, CA 92618-1908, USA; 3 Department of Medicine, University of California Irvine, Irvine, CA 92868, USA

**Keywords:** calcium, cilioplasm, cytoplasm, sensory function, signaling

## Abstract

Primary cilia are hair-like organelles and play crucial roles in vertebrate development, organogenesis, health, and many genetic disorders. A primary cilium is a mechano-sensory organelle that responds to mechanical stimuli in the micro-environment. A cilium is also a chemosensor that senses chemical signals surrounding a cell. The overall function of a cilium is therefore to act as a communication hub to transfer extracellular signals into intracellular responses. Although intracellular calcium has been one of the most studied signaling messengers that transmit extracellular signals into the cells, calcium signaling by various ion channels remains a topic of interest in the field. This may be due to a broad spectrum of cilia functions that are dependent on or independent of utilizing calcium as a second messenger. We therefore revisit and discuss the calcium-dependent and calcium-independent ciliary signaling pathways of Hedgehog, Wnt, PDGFR, Notch, TGF-β, mTOR, OFD1 autophagy, and other GPCR-associated signaling. All of these signaling pathways play crucial roles in various cellular processes, such as in organ and embryonic development, cardiac functioning, planar cell polarity, transactivation, differentiation, the cell cycle, apoptosis, tissue homeostasis, and the immune response.

## 1. Introduction

Cilia are slender microtubule-based sensory organelles that protrude from the apical membrane in many cell types; approximately 800 ciliary proteins have been identified [1,2]. These multifunctional proteins are synthesized in the cytosol and transported to the cilia by intraflagellar transport (IFT) [3]. Cilia sense environmental cues and play vital roles in organ development. Based on their motility, cilia can be classified as motile and non-motile (also referred to as primary cilia). Motile cilia are usually bundled organelles known to be required for mucus clearance, cerebrospinal fluid flow, sperm motility, and leftward flow at the embryonic node, among other functions [4]. Defects in this motility are known to be associated with diseases such as primary ciliary dyskinesia, including Kartagener syndrome. On the other hand, non-motile cilia are solitary sensory organelles. Defects in the sensory function or structure of non-motile cilia are associated with diseases termed ciliopathies, including polycystic kidney. Based on their structure and motility, a special hybrid type of cilia, known as nodal cilia, has been identified. Nodal cilia are solitary motile organelles. Among all types of cilia, nodal cilia are currently known to play the earliest physiological role during embryonic development. Dysfunction in nodal cilia could potentially result in internal thoracic and abdominal organ configuration from normal (situs solitus) to mirror-image configuration (situs inversus), randomized configuration (situs ambiguous), or configuration with duplication (situs isomerism).

## 2. Cilia Structure

**Figure 1:**
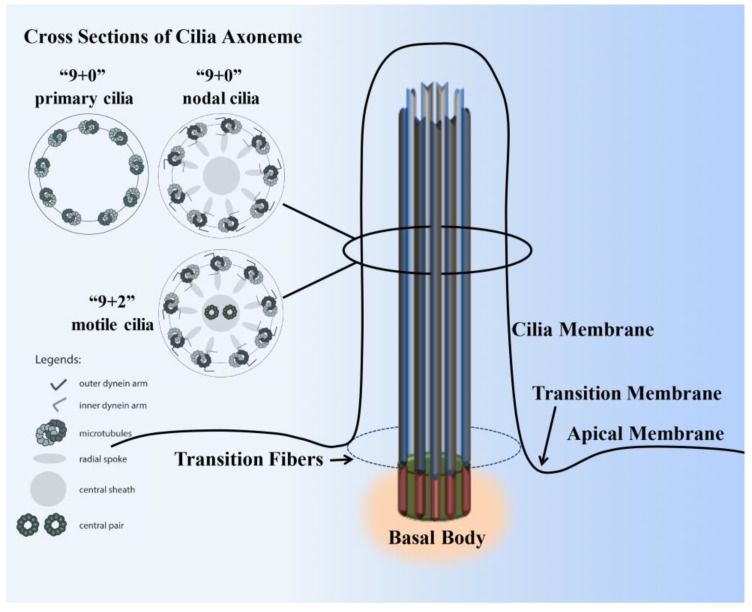
Structure of a cilium. The cilium is a hair-like structure composed of the ciliary membrane, cilioplasm, axoneme, and basal body. This sensory organelle is membrane-bound and contains multiple microtubules running along its length. The ciliary membrane and axoneme make up the upper part of the cilium. The axoneme has nine peripheral microtubule doublets. The axoneme may have two central microtubules (9+2 vs. 9+0 axoneme). The 9+2 cilia usually have dynein arms and radial spokes that link the microtubule doublets and are motile, whereas most 9+0 cilia lack dynein arms, radial spoke, and central sheath and are nonmotile (primary cilia). Some 9+0 cilia only lack the central microtubule and are motile (nodal cilia). The axoneme is enclosed in the ciliary membrane, which is distinct from the cell membrane. Between the cilia and the cell membrane, there is a hypothetical transition membrane surrounding the transition fibers. The transition fibers at the junction of the basal body act as a filter for molecules that can pass into or out of the primary cilium.

The cilium structure contains the microtubule-based cytoskeleton core unit called the axoneme (Figure 1). The axonemal structure is comprised of nine pairs of peripheral microtubules that are post-translationally acetylated to support the long cilia structure. In motile cilia, these nine pairs of microtubules are arranged in circumferentially with a central pair of microtubules (9+2). In the non-motile primary cilia, the axoneme structure usually lacks the central pair of microtubules structure (9+0 arrangement) and many other machineries [5]. In the nodal cilia, the axoneme also lacks the central pair of microtubules (9+0 arrangement), but it requires both dynein arms and radial spokes for motility [6]. The axonemal microtubule structures support the movement of IFT along cilia shaft. IFT includes kinesin-based anterograde and dynein-powered retrograde transport [7,8,9]. A cilium extends from a basal body, which is composed of two centrioles. One of the centrioles is known as the mother centriole, to which the ciliary axoneme is rooted beneath the cell membrane. In addition to its vital structural role, the basal body connected to the transition-fiber proteins is thought to regulate protein entry and exit from the ciliary compartment [10,11]. Protein entering and exiting the cilium is believed to be regulated by a barrier at the ciliary base that encompasses the transition zone (Y-shaped linkers) [12,13]. The Y-shaped linkers, also called Y-ancle, Y-linkers, or simply Y-links, extend from the outer microtubule doublets of the transition zone to the ciliary membrane, generating a very stable membrane domain resistant to detergent [14]. The triplet microtubules at the transition zone are transitioning to doublet microtubules at the ciliary axoneme. The ciliary membrane of the transition zone houses many proteins. The majority of these protein functions are largely unknown and have yet to be established. Some proteins are specifically confined within the transition zone only at a specified time and are later translocated out to the ciliary membrane. For example, many G-protein-coupled receptors are first targeted within the transition zone prior to translocation to the ciliary membrane [15,16,17,18]. The ciliary membrane is therefore known to have many receptor proteins, ion channels, protein transporters and sensory proteins to support the sensory function of the cilia. On the other hand, cilioplasm is constituted within the soluble compartment where many of the signaling proteins are localized.

A general classification of cilia structure is very complex because of the discoveries of a “9+4” axoneme in Hensen’s node of a rabbit and a “3+0” axonemal structure in a protozoan [19,20]. Nonetheless, each part of this cilia structure is crucial to support various signaling molecules. Some of the more established cilia-dependent signaling pathways are described below. Because the roles of primary cilia are diverse, the signaling pathways by cilia are more complex than previously thought. There is currently no literature that summarizes all signaling pathways by primary cilia; in this report, we therefore take the opportunity to provide a brief and concise summary of all known and potential signaling pathways that depend on primary cilia structure and function.

## 3. Calcium Signaling

**Figure 2:**
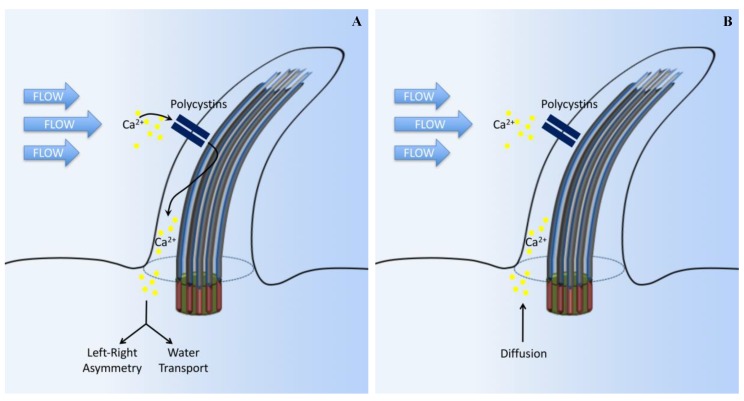
Cilia-dependent calcium signaling. (**A**) The standard established model suggests that primary cilia respond to force through mechanosensation. In this original model, fluid flow bends the cilium, which triggers the opening of calcium-sensitive channel proteins and allows Ca^2+^ to enter the cilium. Intracellular signaling cascades are activated by the Ca^2+^ influx, leading to altered gene expression on the left side of the embryo, or promoting water transport in the kidneys. (**B**) In another model, ciliary bending in response to fluid force does not open Ca^2+^ channels. Instead, Ca^2+^ influx, observed in previous experiments, might have been due to diffusion from the cell body or caused by a damaged cilium membrane in response to extreme levels of shear force.

The association between cilia and calcium (Ca^2+^) signaling has been recognized for decades but had only focused on the motile cilia [21,22]. Recently, many studies have demonstrated that, in different cell types, primary cilia are involved in the regulation of intracellular Ca^2+^ signaling by two major ciliary-associated proteins (polycystin-1 (PC1) and polycystin-2 (PC2)) [23,24,25,26]. In renal epithelial and vascular endothelial primary cilia, PC2 is co-localized with PC1 and functions as a component of a mechanosensory complex [23,24,26]. PC2 activation results in increased intracellular Ca^2+^. The bending of the primary cilium causes cytoskeletal deformation and bending induced membrane stretching at the base of the primary cilium [27]. This, in turn, helps extracellular Ca^2+^ influx through PC2, an event that further stimulates the release of Ca^2+^ from the intracellular stores through the stimulation of ryanodine receptors (Figure 2A) [28]. A more recent advance to study intracellular Ca^2+^ is through the use of genetic markers that specifically encode Ca^2+^-reporter proteins targeted to the cilium. This technique allows for the differentiation of intracellular Ca^2+^ signaling between the cilioplasm and the cytoplasm. Ca^2+^ signals within the cilium mechanically induced by shear stress have also been studied by combining biosensor technology with mechanical flow systems [28,29,30,31]. Using a sensor targeted to primary cilia cells, it is shown that deflecting the cilium with fluid shear force causes an increase in ciliary Ca^2+^ [28,29,30,31].

A study on retinal pigmented cell epithelia demonstrates that changes in the amount of cytosolic Ca^2+^ can occur without a significant alteration in ciliary Ca^2+^, arguing against the hypothesis that channels in the primary cilium would have a significant effect on global cytoplasmic Ca^2+^ [32]. Mechanical rupturing of the cilia membrane led to a rapid increase in ciliary Ca^2+^ propagating to the ciliary base without affecting the cytoplasmic Ca^2+^ level. It is proposed that the cilium can become rapidly infused with tiny amounts of Ca^2+^ that flow in from the cell body (Figure 2B) [33,34,35].

As previously discussed [35], the difference in the intracellular Ca^2+^ models (Figure 2A,B) may be due to genetically-encoded Ca^2+^ indicator (G-GECO) used in the experiments. When changes in ciliary calcium were observed, G-GECO1.0, which has a Kd value of 749 nM, was utilized in these studies [28,29,30,31]. On the other hand, G-GECO1.2, with a Kd value of 442 nM, would have reached saturation of the fluorescent signal intensity by about 90%, resulting in the likelihood of dampening of ciliary calcium [34]. Nonetheless, the question remains of whether a cilium could serve as a mechanosensitive calcium signaling organelle. Of note is that many calcium channels localized in the ciliary membrane are also found elsewhere within the cells. This leads us to question how crucial the difference is between ciliary and cytosolic Ca^2+^ in response to the same stimulus.

Aside from PC2, a primary cilium also contains other transient receptor potential (TRP) channels [35,36,37,38,39]. These TRP channels may also conduct Ca^2+^ signaling. Each transient receptor potential cation channel subfamily member, namely TRPC1, TRPP2, TRPP3, and TRPV4 can be modulated by chemical (Ca^2+^ and receptor ligands), physical (voltage and temperature), and mechanical stimuli (osmolality and fluid shear-stress) [35,36,37,38,39]. Rigorous research on TRPP2 has determined several trafficking mechanisms that regulate transport between the endoplasmic reticulum, plasma membrane, and ciliary membrane [40,41]. In addition, several laboratories independently show that TRPP2 contributes to responses to the mechanical fluid flow in cholangiocytes, embryonic node, left–right asymmetry of zebrafish, smooth muscle cells, and vascular endothelia [23,25,30,42,43,44].

A recent study shows that TRPP3, also known as the PKD2-L1 polycystin ion channel, has atypical Ca^2+^ regulation [39]. This study shows that TRPP3 is partially selective to Ca^2+^ influx. This influx is inhibited by high internal Ca^2+^ concentrations. Moreover, the C-terminal EF-hands and coiled-coil domains of TRPP3 do not contribute to Ca^2+^-induced potentiation and inactivation. It is proposed that the unique characteristics of the TRPP3 enable Ca^2+^ to enter but not leave the small ciliary compartments [39].

Apart from TRP channels, the l-type Ca^2+^ channel also modulates the cystic kidney phenotype, hydrocephalus, and left–right organization defects [45,46]. L-type Ca^2+^ channel knockdown in a zebrafish model facilitates the formation of these ciliopathic phenotypes and the l-type Ca^2+^ channel presence in renal epithelial cilium is confirmed by immunocytochemistry. This confirms the importance of Ca^2+^ signaling in the chemosensory roles of the primary cilium. This l-type Ca^2+^ channel expression also regulates through Wnt signaling [46]. Although the exact mechanism is still not known, it is thought that the modulation of mitochondrial mass and activity results in increased reactive oxygen species that generates oxidative DNA lesions. The subsequent cellular DNA damage response triggers increased CaV1.2 expression [46].

## 4. Hedgehog Signaling

The evolutionarily conserved Hedgehog (Hh) signaling plays an essential role in many aspects of vertebrate embryonic development and stem cell maintenance. Hh signaling is disrupted in a spectrum of human tumors, such as in basal cell carcinoma and medulloblastoma. Although the precise intracellular process of Hh signaling has slightly diverged between species, involving different mechanisms and protein components, primary cilia have an essential role in Hh signaling in both vertebrate and invertebrate development [47,48]. The association between Hh and cilia was first established from genetic screening in the mouse based on the neural tube patterning of mouse embryos. This screening identifies a set of genes that are required for normal Hh signaling and the formation of primary cilia.

**Figure 3:**
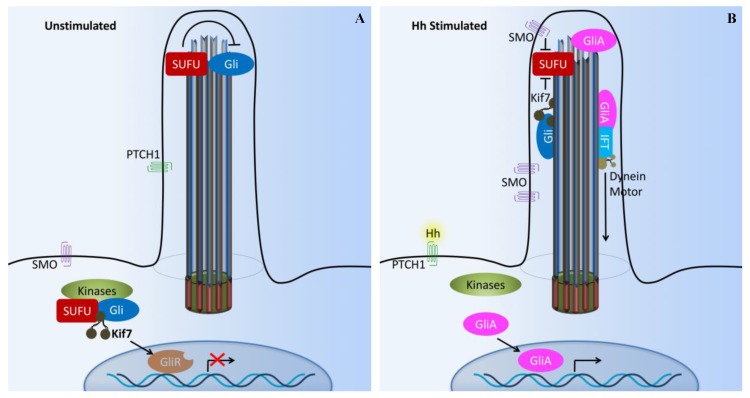
Cilia-dependent Hedgehog signaling. (**A**) The Hedgehog (Hh) pathway in vertebrates utilizes cilium as a signal transduction compartment. In the absence of the Hh ligand, the PTCH1 receptor localizes to the cilium and is thought to block the entry of SMO into cilia. The kinesin Kif7 (the COS2 homologue) localizes to the base of the cilium, where it may form a complex with Gli transcription factors and other pathway components. Kif7 at the cilium base prevents Gli proteins’ enrichment within the cilium and promotes processing of GliRs. (**B**) Upon Hh binding, SMO moves to the ciliary membrane and Kif7 translocates into the primary cilium, thereby promoting Gli accumulation at the ciliary tip. Kif7 and SMP in the cilia may also block the function of SUFU. Activated Gli (GliA) is transported out of the cilium by the dynein motor and intraflagellar transport particles (IFT). X and T symbols denote inhibition of gene transcription and protein function, respectively.

The cilium has been suggested to function as the Hh transduction hub [49]. Hh signaling involves multiple inhibitory interactions. In the absence of Hh ligand (Figure 3A), Hh transmembrane receptor Patched1 (PTCH1, Hh 12-transmembrane receptor) is located at the base of a primary cilium and inhibits the activity of seven transmembrane smoothened proteins (SMO) through an unidentified signaling cascade. Meanwhile, suppressor of fused (SUFU) and three zinc-finger-containing transcriptional factors (Gli1, Gli2, and Gli3) interact with each other within the cilium. This leads to the inhibition of Gli transcriptional repressor (Gli3) formation and stimulation of Gli transcriptional activators (Gli1 and Gli2) [47,50]. Thus, the transcriptional activation by Hh is largely driven by Gli1 and Gli2.

In the presence of an Hh ligand (Figure 3B), PTCH1 is activated. This triggers PTCH1 to move out of the cilium and releases the inhibition of SMO, allowing SMO translocation into the cilium [51,52]. Activated SMO co-receptor translocates into the cilium and activates Gli (Gli activator) by releasing the inhibition of SUFU. Furthermore, the Gli activator is transported to the cell nucleus to turn on the expression of other genes [53,54,55]. The repressor complex composed of SUFU and kinesin-like KIF7 sequesters Gli2 and Gli3 in the cilium tip. Sequential phosphorylations of Gli2 and Gli3 by protein kinase A (PKA), glycogen 3b (Gsk3b), and casein kinase 1 (Ck1) create a phophopeptide motif on Gli2 and Gli3 that is recognized by Skip/Cullin1/F-box E3 ubiquitin ligase β-TrCP (β-transducin repeat-containing protein) [56]. The addition of ubiquitin then directs the Gli proteins to the proteasome for limited (Gli3) or complete (Gli2) degradation by the proteasome. This in turn regulates the abundance of the Gli2 and Gli3 repressor forms that ultimately inhibit Hh target genes in the nucleus [57,58,59,60]. Inhibition of Gli3 and activation of Gli2 in response to Hh are largely dependent on cilium function [47,61,62].

## 5. Wnt Signaling

**Figure 4:**
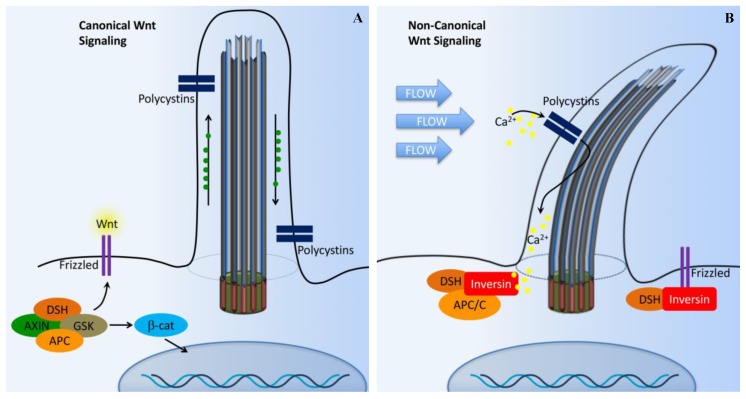
Cilia-dependent Wnt signaling. The cilia/basal body may function as a regulatory switch to control the canonical and non-canonical Wnt signaling pathways. (**A**) In the absence of fluid flow, canonical Wnt signaling predominates. Wnt ligand binds to the co-receptors frizzled, dishevelled (DSH) is recruited to frizzled, and glycogen synthase kinase-3 (GSK3) is inactivated. β-catenin (β-cat) translocates to the nucleus, where it acts as a transcriptional co-activator with members of the LEF and TCF family and induces transcription of Wnt target genes such as cMYC, AXIN2, or L1CAM. (**B**) In non-canonical Wnt signaling, the mechanosensation by fluid flow causes intracellular Ca^2+^ increase and an increase inversin expression. Inversin resides in multiple locations in the cell as well as at the base of the cilium. Inversin targets cytoplasmic DSH for anaphase-promoting complex/cyclosome (APC/C)-dependent ubiquitylation and degradation, making it unavailable for canonical Wnt signaling.

Another important pathway required for ciliary protein transportation for normal regulation is Wnt pathways (canonical and non-canonical; Figure 4) [63]. The Wnt signaling pathway is evolutionarily conserved in many species, ranging from flies to human, and the signaling is mediated within primary cilia. Wnt signaling is involved in cell migration, planar cell polarity, neural patterning, skeletal system development, and organogenesis. In cultured cells and zebrafish embryos, ciliary proteins have been shown to regulate both non-canonical and canonical Wnt signaling pathways [64,65,66,67]. Wnt binding to membrane-bound receptors (frizzled) can activate both canonical (β-catenin-dependent) and non-canonical (β-catenin-independent) Wnt signaling.

In the presence of a canonical Wnt signal (Figure 4A), cytosolic levels of β-catenin are high due to the inhibition of the β-catenin destruction complex [55,63,68,69]. This complex is composed of Axin, adenomatous polyposis coli (APC), casein kinase 1 (CK1), and glycogen synthase kinase 3β (GSK-3β). The β-catenin destruction complex operates within the β-TrCP/SCF-dependent ubiquitin–proteasome pathway. In the absence of Wnt, the phosphorylation of β-catenin by CK1 and GSK-3β is thought to act as a trigger for degradation. This process occurs at the basal body. Furthermore, disheveled (DSH) regulates canonical Wnt signaling through direct inhibition of GSK-3β kinase activity [70].

In the presence of Wnt, the β-catenin destruction complex is anchored in the plasma membrane by DSH, resulting in the inactivation of the complex. As a result, the β-catenin level is increased in the cytoplasm. β-catenin then translocates into the nucleus to function as a transcriptional coactivator. β-catenin associates with the nuclear transcription factors T-cell factor (TCF) and lymphocyte enhancer factor (Lef), which interacts with various transcriptional suppressors, such as Groucho.

The non-canonical Wnt pathway is less well characterized. It is thought that the non-canonical pathway is independent of β-catenin, but dependent on DSH (Figure 4B). The non-canonical Wnt signaling is involved in controlling tissue functions and maintaining tissue architectures by modulating cell migration and orientation. The control of cells in the plane of a tissue, also known as planar cell polarity (PCP) pathway, was first established in *Drosophila* [71]. The mechanosensation-dependent calcium (Ca^2+^) pathway is believed to be an important initiator for non-canonical Wnt signaling. DSH is recruited to the plasma membrane to prevent the proteasomal degradation of DSH in a β-catenin-independent manner. Several studies have implicated many other ciliary and basal body proteins in the regulation of Wnt signaling [67,72,73,74]. Inversin, in particular, interacts with DSH and targets the cytoplasmic fraction of DSH for degradation. Inversin is thought to function as a molecular switch required for non-canonical Wnt signaling and the suppression of β-catenin activity [67]. Inversin thus regulates the balance between the canonical and non-canonical Wnt signaling. Notably, inversin also functions to separate protein pools among the basal body or cilium, cytoplasm, adherens junction, and nuclear proteins [67,75]. However, it is currently unknown how inversin navigates its role among those protein pools.

A recent study shows that several Wnt ligands can bind to PKD1 and that PKD1/TRPP2 can mediate the effects of other Wnts, not only in the kidney but also in other organs where Wnt/Ca^2+^ activity is present [76]. This study further suggests that the PKD1/TRPP2 channel complex can function as a ligand-activated channel. Specifically, Wnt3A, a typical canonical Wnt, activates TRPP2, suggesting that Wnts could induce Ca^2+^ influx regardless of their ability to signal through β-catenin as long as they can bind to PKD1 and PKD1/TRPP2 is present in the target cell.

## 6. PDGFR Signaling

**Figure 5:**
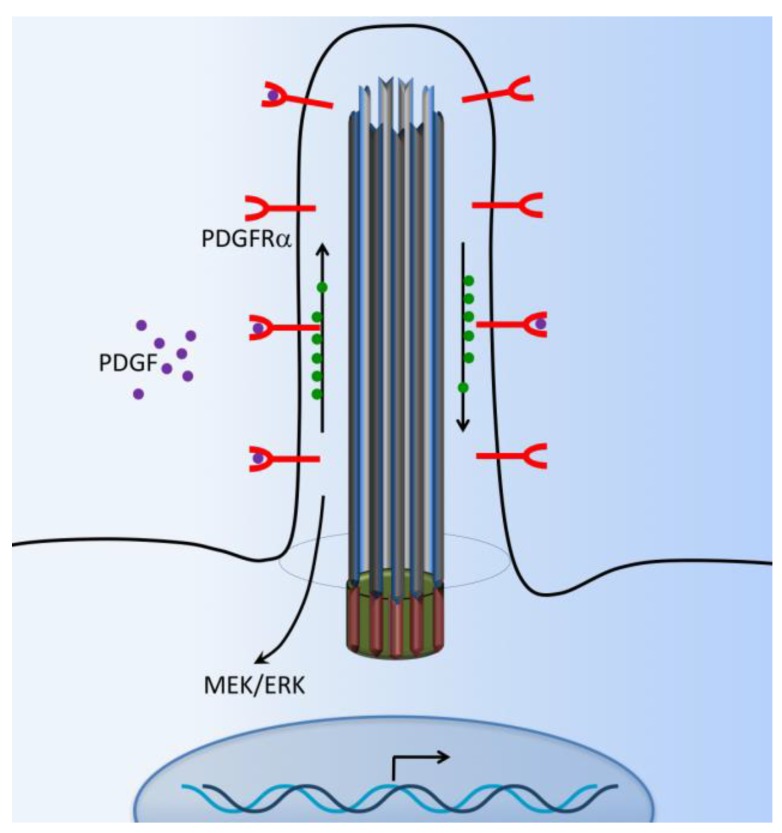
Cilia-dependent PDGFR signaling. The G-protein coupled receptors such as platelet-derived growth factor receptor (PDGFRα) are located at the cilia membrane. The PDGFR pathway is initiated with PDGF ligand binding to their receptors and inducing cellular responses through downstream signaling pathways such as the MEK/ERK cascade.

Platelet-derived growth factor (PDGF) and their receptors (PDGFRs) play pivotal roles in cell survival, growth control, proliferation, cell migration, embryonic development, and the maintenance of tissue growth. The PDGFR is a receptor tyrosine kinase that mediates signaling events through primary cilia [77]. Abnormal PDGF signaling causes a range of diseases, including cancer development, cardiovascular inflammation, or fibrosis [78]. PDGFRα-signaling is coordinated by the primary cilium in mouse cultured embryonic fibroblasts [79,80]. PDGFRα is a tyrosine–kinase receptor located within the ciliary membrane. In normal fibroblasts and the fibrosarcoma cell line, the binding of ligand PDGF-AA activates the dimerized PDGFR-αα receptor, which is present in the extracellular flow, and induces downstream cellular responses via MEK/ERK cascade signaling pathways (Figure 5). These pathways specifically lead to cellular growth, cytoskeletal development, and cellular migration and their differentiation [55,81,82]. Subsequently, PDGFRα-signaling through the fibroblast primary cilium may also be important in tissue homeostasis, while defects in this pathway could lead to tumorigenesis [83]. It has been reported that β-catenin may form a complex with PDGFRα to modulate cell migration [82].

## 7. Notch Signaling 

Notch signaling plays a key role in various aspects of patterning and cell fate choice in neurogenesis and maintenance of adult tissue growth and development. Notch signaling balances progenitor cells with differentiated neurons; it regulates the binary cell fate choice of progenitors as they segregate into different neuronal subtypes [84,85,86,87,88,89]. In mammals, Notch refers to four different kinds of receptors, Notch1–4. The Notch receptor has a single transmembrane domain with a large extracellular portion associated with Ca^2+^ and a short intracellular portion. There is evidence suggesting that some Notch signaling may depend on primary cilium for the signal transduction [90,91].

**Figure 6:**
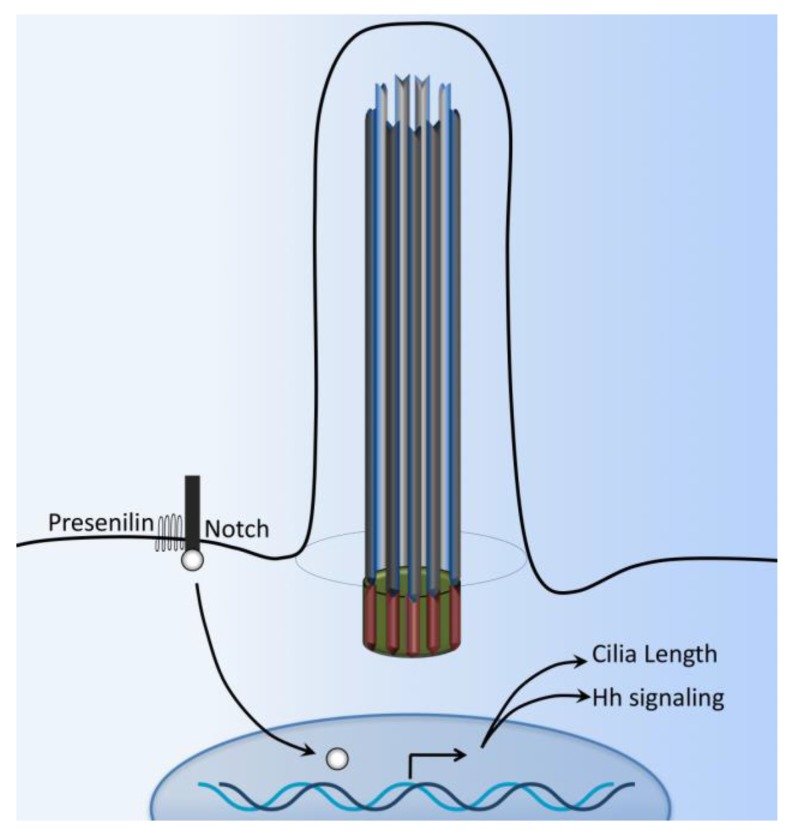
Cilia-dependent Notch signaling. A single-transmembrane Notch forms a receptor complex with the eight-transmembrane presenilin. Upon activation, presenilin mediates the intramembranal cleavage of notch containing the cdc10/Ankyrin repeats. The Notch intracellular domain is then released to the nucleus to activate various gene transcriptions.

The Notch3 receptor interacts with Presenilin-2, an enzyme responsible for Notch cleavage and activation (Figure 6). The Notch3 receptor has also been shown to localize in the ciliary membrane while Presenilin-2 is localized to the ciliary basal body [92]. Zebrafish with defects in basal body proteins (such as bbs1 and bbs4) have an increased expression of Notch targets. In these zebrafish models, the Notch receptor accumulates in the late endosomes, and consequent lysosomal degradation of Notch receptor is impaired [90]. The Notch signaling pathway plays a central role in left-right asymmetry by regulating cilium length and thus plays a crucial role in cilium length control [93,94]. Finally, Notch signaling also promotes trafficking of the sonic hedgehog (Shh) signaling mediators into primary cilium, thereby enhancing the responsiveness of neural progenitor cells to Shh [95].

## 8. TGF-β Signaling

**Figure 7:**
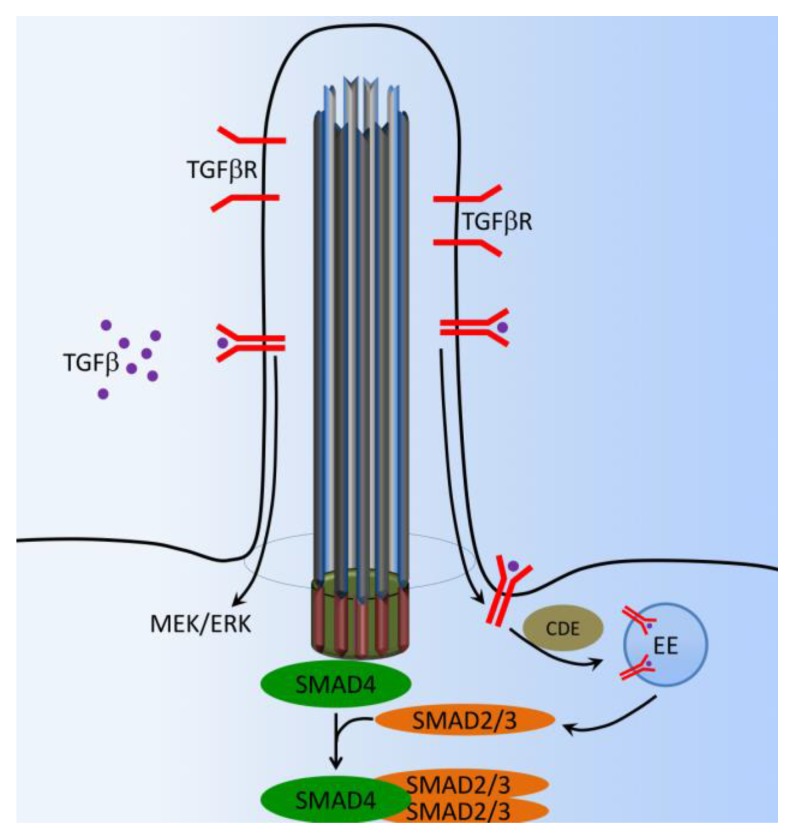
Cilia-dependent TGF-β signaling. In the presence of TGF-β, activated TGF-β receptors (TGF-β1 and TGF-β2) translocate from the ciliary membrane to the ciliary pocket (CiPo) for clathrin-dependent endocytosis (CDE) and activation of SMAD transcription factors 2/3 (SMAD2/3) in early endosomes (EE) at the ciliary base. SMAD2/3 forms a trimeric complex with SMAD4 for nuclear translocation and activation of TGF-β target genes. Ciliary TGF-β signaling also involves the activation of ERK1/2.

Transforming growth factor beta (TGF-β) plays major roles in bone development and maintenance metabolism by regulating cellular proliferation, differentiation, matrix deposition, and cell migration [96,97]. TGF-β1/Bone morphogenic protein (BMP), which is localized in the extracellular bone matrix, is activated during osteoclast-mediated resorption. The TGF-β1/BMP complex is released in response to loading or injury such as a bone fracture [98,99,100,101]. Recent studies have elegantly demonstrated that a discrete spatial group of TGF-β1 and TGF-β2 receptors is localized within and around the primary cilium in embryonic fibroblast and stem cells [102,103,104]. Both TGF-β1 and TGF-β2 receptors localize to the ciliary tip and endocytic vesicles at the ciliary base (Figure 7). TGF-β stimulation increases clathrin-mediated receptor localization and activation of SMAD2/3 and ERK1/2 at the ciliary base, which is increased in stem cells undergoing cardiomyogenesis [104]. TGF-β1-induced recruitment of human mesenchymal stem cells (MSCs) is regulated by the primary cilium. Furthermore, the cilium mediates this response through distinct spatial localization of TGF-β receptors and signaling components at the primary cilium, whereby TGF-β1-mediated activation of SMAD3 takes place at the ciliary base (Figure 7). Signaling through the primary cilium is necessary for the sensing of a TGF-β1 chemotactic gradient in human bone MSCs, demonstrating a novel mechanism of TGF-β signal regulation and recruitment in stem cells. This highlights the primary cilium as a possible target to enhance MSCs recruitment and bone formation in orthopedic disease [105].

## 9. mTOR Signaling

**Figure 8:**
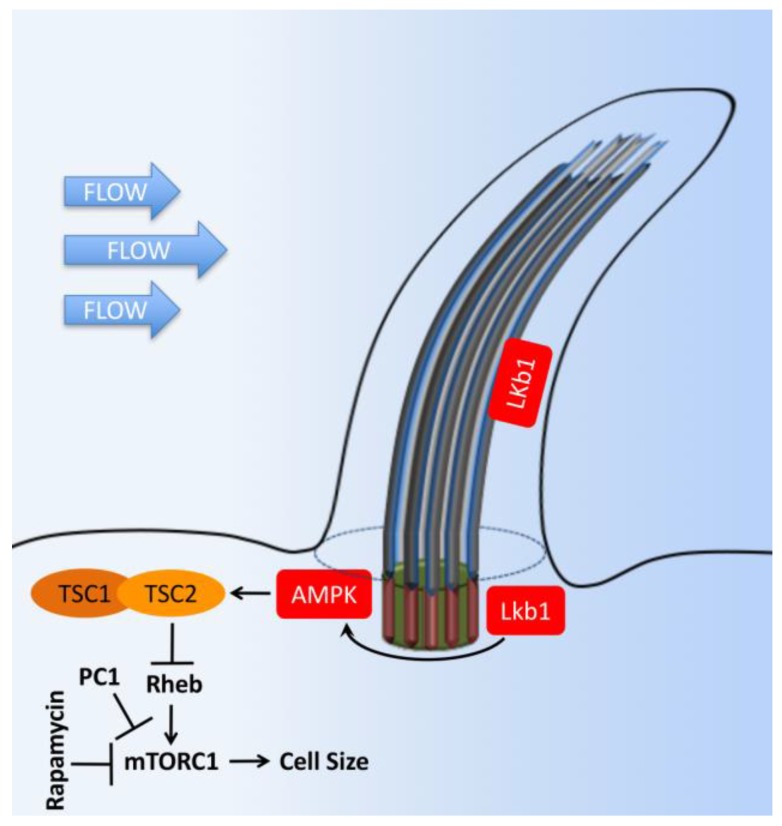
Cilia-dependent mTOR signaling. Flow shear stress mediates the bending of primary cilia and inhibition of mTORC1. Tumor suppressor kinase (LKB1) and AMP-dependent protein kinase (AMPK) are found in the cilia and basal body. Upon cilium bending by fluid flow, LKB1 is activated and transported into the basal body, where AMPK is phosphorylated. Furthermore, AMPK phosphorylates TSC2 to activate the TSC1–TSC2 complex. In turn, TSC1–TSC2 stimulates the GTPase activity of Rheb, preventing it from activating mTORC1. Otherwise, AMPK could also directly antagonize mTORC1. Polycystin-1 (PC1) may also exert its inhibitory effect on the mTORC1 pathway through its known interaction with TSC2. The T bars denote inhibition of protein function.

Mammalian target of rapamycin (mTOR) is a serine/threonine protein kinase functioning as a central regulator in cellular metabolism, cell growth, cell proliferation, cell cycle progression, and survival. mTOR associates with different proteins to form the rapamycin-sensitive (mTORC1) and -insensitive (mTORC2) complexes, respectively. mTOR signaling and primary cilia are associated with the hyper-proliferation of renal tubular cells, which induces the formation of renal cysts [106]. The most intensive studies about the primary cilium and mTOR in kidney research lie in the polycystic kidney-associated proteins [107]. The mTOR subcellular signaling involves many proteins, and the detailed subcellular localization of mTOR family members is largely unclear except that Hamartin (TSC1) is localized to the base of the primary cilium (Figure 8) [108]. In a recent study, the LKB-1-AMPK-mTOR signaling pathway is proposed in the kidney for the flow-dependent downregulation of the mTOR pathway. This pathway is necessary for the proper control of cell size by autophagy [109]. It is proposed that the primary cilia prevent cyst progression and kidney tubular cell hypertrophy and proliferation [110], and PC1 inhibits mTOR activity through the regulation of tuberous sclerosis complex (TSC2) localization [111].

## 10. OFD1 and Autophagy Signaling

**Figure 9:**
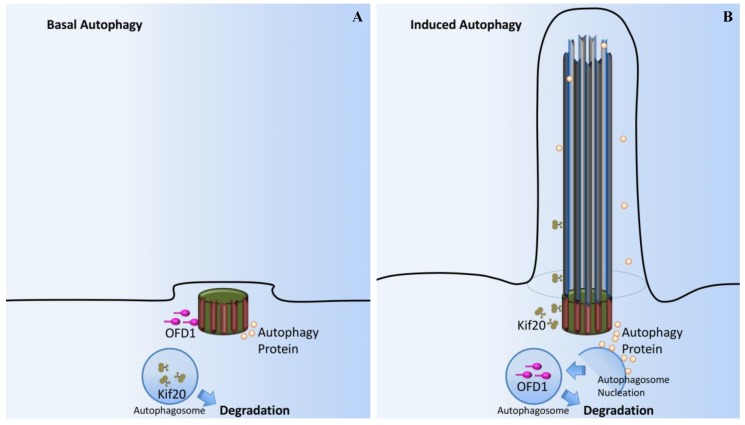
Cilia-dependent OFD1/Autophagy signaling. (**A**) In basal autophagy, ciliogenesis is prevented by the sequestering of IFT20 in autophagosomes and its autophagic degradation. OFD1, a protein that constrains cilium formation at centriolar satellites, is not affected by basal autophagy and ciliogenesis is therefore impaired. (**B**) Autophagy induced by serum deprivation promotes ciliogenesis through inactivation of OFD1, while sparing IFT20. Autophagy proteins are also confined to this sensory organelle, probably contributing to cilium organization. At the basal body, signals from the cilium stimulate autophagosome nucleation and OFD1 degradation.

Autophagy is a process by which cells break down cytoplasmic contents. Oral facial digital syndrome 1 (OFD1) localizes to centrioles and centriolar satellites (Figure 9A). At the basal autophagy level, OFD1 and the autophagosome marker LC3 are co-localized at the basal body [112]. In starvation-induced autophagy, OFD1 is degraded (Figure 9B); the decreased OFD1 level upon serum starvation is contingent on both the autophagy-related (ATG) protein ATG5 and lysosome function. Fascinatingly, OFD1 degradation is specific to the centriolar satellite pool of this protein as OFD1 levels at the centrioles remain unchanged following serum starvation. OFD1 at the centriolar satellites suppresses ciliogenesis and that starvation-induced autophagy removes OFD1 from centriolar satellites.

Another study explores the effect of ciliogenesis on autophagy [113]. Starvation-induced autophagy and autophagosome biogenesis are dysfunctional in cell lines with abnormal IFT20 or IFT88, proteins required for cilia formation. Additional experiments show that signaling by the Hh pathway, components of which are recruited to the cilia by IFT, is required for cilia-induced autophagy. Increasing Hh signaling tends to induce autophagic flux, while decreasing Hh will prevent autophagy. This mechanism is abnormal in IFT20 or IFT88 mutants. These data suggest that primary cilia promote autophagosome formation.

## 11. Other Cilia-Dependent Signaling Pathways

G-protein coupled receptors (GPCRs) constitute a substantially diverse family of seven-transmembrane receptors that transmit specific signals to the cell through G proteins that regulate the activity of various cellular and physiological signaling networks. GPCRs also localize to rodent neuronal cilia, including the serotonin subtype-6 receptor (5-HTr6), somatostatin receptor3 (SSTR3), and melanin-concentrating hormone receptor 1 (MCHR1) [18,114,115]. Although receptors were found on neuronal cilia a long time ago, the functional roles of these GPCRs are still elusive.

The elusive role of ciliary kisspeptin receptor (KISS1R) in the onset of puberty and adult reproductive function is observed in gonadotropin-releasing hormone (GnRH) neurons [116]. While the selective ablation of cilia from GnRH neurons affects neither cell migration nor sexual maturation in the mice, the loss of the cilia in GnRH neurons seems to alter neuronal signaling. All these findings demonstrate that ciliary localization of a GPCR can have different effects on cellular metabolic function compared to being distributed throughout the membrane.

Overexpression of the serotonin subtype-6 receptor (5-HT6) in cilium induces abnormally long cilia in rat brains that also have significantly reduced dendritic branches [117]. This effect may be associated with signaling through type 3 adenylyl cyclase (AC) and important ciliary AC and cAMP, which have been found to impact cilia length and further control neuronal cell development [118].

Another GPCR that has been associated with cilia signaling is dopamine receptors. Dopamine is a circulating hormone that plays a fundamental role as a neurotransmitter. Dopamine has been implicated in hypertension in humans and animal models. The roles and functions of dopamine vary in different tissues in the body. The renovascular dopaminergic system is involved in renal blood flow and blood pressure control, but its specific regulation is not yet known. Genetic screening for abnormal cilia length has identified a family of class-A dopamine binding GPCRs that are involved in the regulation of cilia and flagella length [119]. One of the many regulators of cilia length is the orphan GPR22, a rhodopsin-like GPCR that couples to Gαi/αo to inhibit AC. GPR22 regulates cilia length and structure as well as left–right asymmetry in zebrafish [120]. The expression of the dopamine receptor in renal epithelial and vascular endothelial primary cilia has further led to the hypothesis in the mechanism and signaling of dopamine involved cilia-specific signaling [121,122]. Activation of the ciliary dopamine receptor induces vasodilation via changes in the chemical and mechanical properties of primary cilia through changes in the cilia length. Another regulator of cilia length is oculocerebrorenal syndrome of Lowe 1 (OCRL1), a lipid phosphatase that has been shown to modulate cilia length in renal epithelial cells. OCRL1 knockdown leads to elongation of cilia and reduced intracellular Ca^2+^ release in response to ATP [123].

## 12. Conclusions and Perspectives

It is important to note that while the cellular and molecular pathways described above have been studied in the context of cilia structure or function, there is evidence that some pathways could also take place independent of cilia. For example, it has been suggested that cilia are not involved in Wnt signaling [124,125]. When a Wnt ligand binds to its receptor, the effector β-catenin translocates to the nucleus [126,127]. Although the roles of many ciliary proteins have been shown to alter the translocation of β-catenin to the nucleus [63,65], there is contradicting evidence about the roles of cilia on the Wnt signaling, ranging from inactivation to overactivation of the pathway [128]. Likewise, Hedgehog (Hh) signaling has also been shown to function independently of cilia [129]. It is shown that Hh regulation of Gli protein levels by Sufu is cilium-independent [129], despite the fact that many Hh pathway components are localized to cilia. It is therefore conceivable to assume that the cellular signaling described above could mechanistically involve both cilium-dependent and cilium-independent signal transduction pathways.

In summary, the primary cilium remains a vital organelle responsible for integrative signaling from extracellular signals in order to exert the physiological functions within a cell. It would certainly be a generalization to assume that a single type of cilium would serve most of the needs of every cell. The key to our understanding of cilia function in cellular signaling might be to differentiate the roles of cilia in different cells, tissues, and organs. Although abnormal ciliary proteins cause ciliopathies, which present a diversity of clinical features, we do not have a clear idea as to how the cilia signaling compartment plays an ultimate role in the pathogenesis. There needs to be further research on primary cilia as this neglected organelle continues to disclose its internal mysteries.

## Supplementary Materials

Supplementary materials can be found at www.mdpi.com/1422-0067/18/11/2272/s1.
